# Synergic Anti-Pruritus Mechanisms of Action for the *Radix* *Sophorae Flavescentis* and *Fructus Cnidii* Herbal Pair

**DOI:** 10.3390/molecules22091465

**Published:** 2017-09-04

**Authors:** Jiali Zhong, Zhihong Liu, Xinxin Zhou, Jun Xu

**Affiliations:** 1 School of Chinese Materia Medica, Guangzhou University of Chinese Medicine, Guangzhou 510006, China; 18702011874@163.com; 2 School of Pharmaceutical Sciences, Sun Yat-Sen University, 132 East Circle Road at University City, Guangzhou 510006, China; zhihongliu2011@gmail.com

**Keywords:** *Radix Sophorae Flavescentis* (RSF), *Fructus Cnidii* (FC), anti-pruritus, synergic mechanism

## Abstract

*Radix Sophorae Flavescentis* (RSF) and *Fructus Cnidii* (FC) compose a typical herbal synergic pair in traditional Chinese medicine (TCM) for pruritus symptom treatments. The mechanisms of action for the synergy are not understood. This paper aims at predicting the anti-pruritus targets and the main active ingredients for the RSF and FC herbal pair. We demonstrate that the RSF–FC herbal pair can be elucidated by mining the chemical structures of compounds derived from RSF and FC. Based on chemical structure data, the putative targets for RSF and FC were predicted. Additional putative targets that interact with the anti-pruritus targets were derived by mapping the putative targets onto a PPI network. By examining the annotations of these proteins, we conclude that (1) RSF’s active compounds are mainly alkaloids and flavonoids. The representative putative targets of the alkaloids are inflammation-related proteins (MAPK14, PTGS2, PTGS2, and F2) and pruritus-related proteins (HRH1, TRPA1, HTR3A, and HTR6). The representative putative targets of the flavonoids are inflammation-related proteins (TNF, NF-κB, F2, PTGS2, and PTGS2) and pruritus-related proteins (NR3C1 and IL2). (2) FC’s active compounds are mainly coumarins. Their representative putative targets are CNS-related proteins (AChE and OPRK1) and inflammation-related proteins (PDE4D, TLR9, and NF-κB). (3) Both RSF and FC display anti-inflammatory effects, though they exhibit their anti-pruritus effects in different ways. Their synergy shows that RSF regulates inflammation-related pruritus and FC regulates CNS-related pruritus.

## 1. Introduction

Pruritus, especially when associated with chronic diseases such as atopic dermatitis, can be difficult to cure in the clinic and has a major impact on the quality of life of patients. Inflammation, dry skin, immune disorders, and many pathogens can cause pruritus symptoms [1]. Currently, therapeutic agents, such as glucocorticoids, cyclosporin A, and tacrolimus, can only partially meet patient needs [2,3,4]. Topical corticosteroids have many side effects such as skin atrophy [5]. New anti-pruritus agents without side effects are therefore in demand.

With its long history, traditional Chinese medicine (TCM) has accumulated significant anti-pruritus prescription data [6]. One of the most important TCM treatment characteristics involves herbs used in pairs. Herbs in a pair must be compatible and work together against specific pruritus symptoms [7]. The relationships between a herb pair can be mutual enhancement, assistance, or complementary [8]. Herbs can also work against one another, though we will not discuss this relationship in this paper.

*Radix Sophorae Flavescentis* (RSF) and *Fructus Cnidii* (FC) belong to a classic antipruritic herb-pair in TCM. RSF, the dried roots of *Sophora Flavescens* Aiton, was first recorded in “Shennong Bencao Jing” 2000 years ago [9]. RSF has diuresis, detoxification, expelling dampness, and insect repellent functions [10]. RSF is also one of the major ingredients in a Chinese Food and Drug Administration (CFDA)-approved topical vaginal medication (Jie Er Yin) for the treatment of contact dermatitis and local pruritus [11]. Recent studies have demonstrated that RSF has many other pharmacological effects including anti-inflammation, antibacterial, analgesic, anti-tumor, and anti-hepatitis B virus [12,13]. However, the evidence is not sufficient to recommend matrine for routine clinical use yet due to the low methodological quality of the studies. Further rigorous trials are needed [9,14,15].

FC is the dried ripe fruit from *Cnidium monnieri* (L.) Cusson. It has functions in warming kidney tissue, promoting virility, removing dampness, dispelling wind, and repelling parasites [16]. FC is commonly used for anti-pruritus in the genitalia, and for curing suppurative dermatitis. Pharmacological studies showed that FC does have anti-allergic, androgenic disorder, antibacterial, and antipruritic effects [17,18].

The RSF and FC herbal pair are commonly used as a topical medication against pruritus and are the main ingredients in many CFDA-approved drugs (such as, the TCM drugs listed in http://eng.sfda.gov.cn/WS03/CL0755/). However, the mechanism of action of the herbal pair remains unclear because the TCM prescription contains many chemical compounds and the ligand–target relationships involve sophisticated networks [19,20,21,22,23,24].

**Figure 1:**
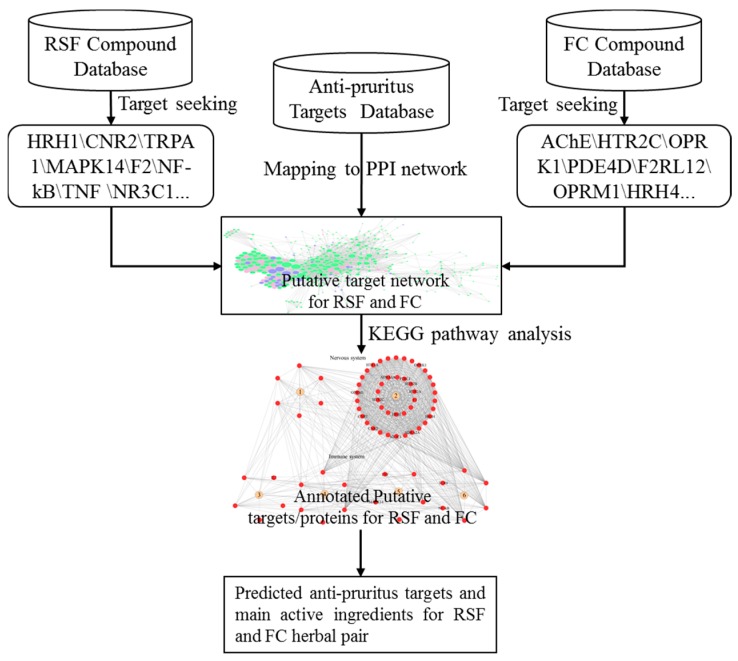
Flow chart for elucidating the mechanisms of action for the synergy of the RSF–FC herbal pair against pruritus.

To investigate the synergic mechanism of RSF and FC for anti-pruritus, we collected the active ingredient data and associated target information for RSF and FC from publications. A herb-scaffold-target network was constructed based on the data. Since the predicted and literature-reported anti-pruritus targets are not completely consistent, we selected the intersections of the two target sets as putative anti-pruritus targets. With a protein–protein interaction network, we identified major hub nodes as significantly associated with anti-pruritus targets. This study proposes a protocol to identify the synergic mechanism of RSF and FC. The protocol (Figure 1) aims to investigate the potential synergistic mechanism of RSF and FC compounds.

## 2. Results

### 2.1. Putative Target Prediction of RSF and FC

To predict the putative targets of RSF and FC, we derived molecular scaffolds from the active components of RSF and FC. By comparing the scaffolds of herbs against the ligands of known targets, we identified 321 known targets of 32 molecular scaffolds from the Annotated Scaffold Database (ASDB) [25]. The other 28 molecular scaffolds have 285 putative targets, which are predicted by the Pharmaceutical Target Seeker (PTS, http://www.rcdd.org.cn/PTS/index) with a 3D structure similarity threshold of 90% and a ligand activity threshold (IC_50_/Ki/Kd) <10 μM. Thus, a total of 494 putative targets of 57 molecular scaffolds (3 molecular scaffolds, such as flavone, chromone, and simple coumarin, derived from both RSF and FC) were studied with respect to RSF and FC in this article.

### 2.2. The Relationship among the Herbs, Ingredients, and Targets of RSF and FC

**Figure 2:**
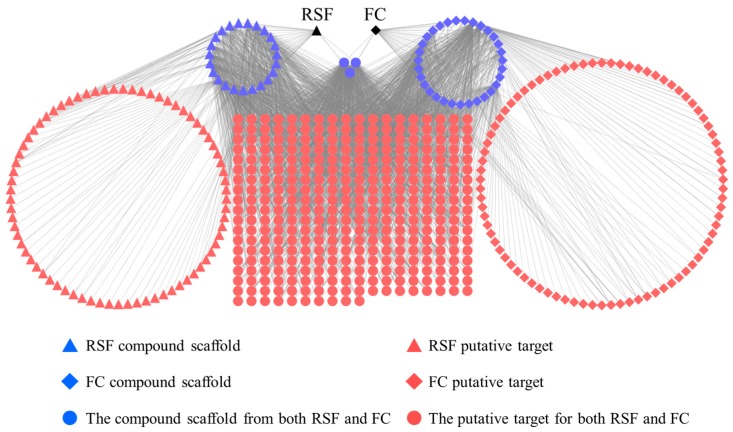
Herb-scaffold-target network. The black nodes represent herbs, the blue nodes represent scaffolds, and the red nodes represent putative targets. RSF, *Radix Sophorae Flavescentis* and FC, *Fructus Cnidii*.

TCM exerts biological and pharmacological effects through multiple compounds that regulate multiple targets. These relationships are demonstrated in an herb-scaffold-target network as shown in Figure 2. This network consists of 553 nodes (2 herbs, 57 candidate scaffolds and 494 putative targets) and 2599 compound–target interactions. The average degree of the nodes (molecular scaffolds) is 45.5, of which 21 molecular scaffolds possess degrees greater than 46. These scaffolds are more likely to regulate multiple targets. Alkaloids and flavonoids are the main RSF moieties [26,27]. In RSF, matrine and oxymatrine (alkaloids) are active components, and are associated with 84 putative targets. (2r)-flavanone and isokurarinone (dihydrogen flavonoids) from RSF are associated with 61 targets. In FC, coumarins are the main components [16]. The coumarins (such as osthole, ostruthin, and isomexoticin) in FC are associated with 92 putative targets. The biological activities of RSF and FC were identified by means of network analyses (Supplementary Table S1).

Many targets are associated with multiple compounds in the network. Thrombin (F2), prostaglandin G/H synthase 1 (PTGS1), and nuclear factor-kappa-B p105 subunit (NF-κB) are potentially regulated by 14 (representing 21 compounds), 13 (representing 55 compounds), and 10 (representing 49 compounds) scaffolds derived from RSF or FC. These targets are responsible for inflammation and immune system activation [28,29,30].

RSF and FC have 335 common putative targets, 159 uncommon putative targets, and 3 common molecular scaffolds that associate with common putative targets.

### 2.3. Putative Anti-Pruritus Targets of RSF and FC

Anti-pruritus targets were derived from the Therapeutic Targets Database (TTD) [31] by using the keyword “pruritus”. The anti-pruritus targets included histamine H1 receptor (HRH1), 5-hydroxytryptamine 3 receptor (HTR3), proteinase activated receptor 2 (F2RL1), vanilloid receptor 1 (TRPV1), chlorine/potassium channel receptor, and 5-hydroxytryptamine 6 receptor (HTR6). By reviewing the literature [32,33,34,35,36,37], we summarized 81 pruritus-related targets (including the above-mentioned targets), which are the myriad of mediators capable of stimulating pruritus sensory nerve terminals (includes both unmyelinated C-fibers and thinly myelinated Ad nerve fibers) leading to itch, including biogenic amines, proteases, cytokines, and peptides. (Supplementary Table S2).

**Figure 3:**
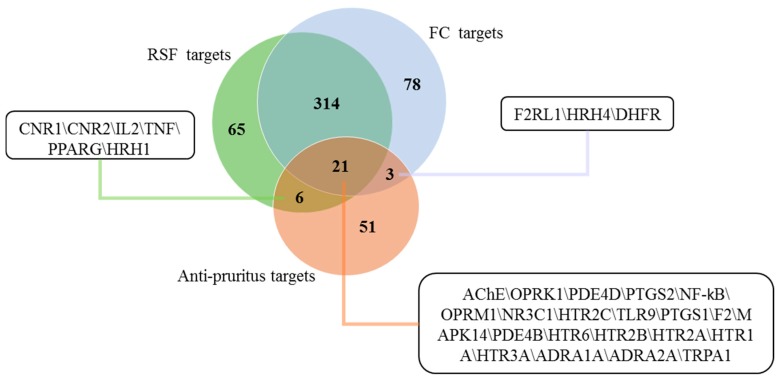
Putative anti-pruritus targets of RSF and FC. Venn diagram for putative targets of RSF and FC and known anti-pruritus targets. The list of targets in the intersection sets.

As shown in Figure 3, the three target sets, anti-pruritus targets (orange), putative targets for RSF (green), and putative targets for FC (blue), have intersections containing 30 targets. Among the intersected targets, 21 targets are at the intersections of three target sets, 6 targets are at the intersections of the green and orange sets, and 3 targets are at the intersection of the blue and orange sets. The 30 targets were considered as putative anti-pruritus targets.

### 2.4. Interactions between Putative Targets and Anti-Pruritus Targets

**Figure 4:**
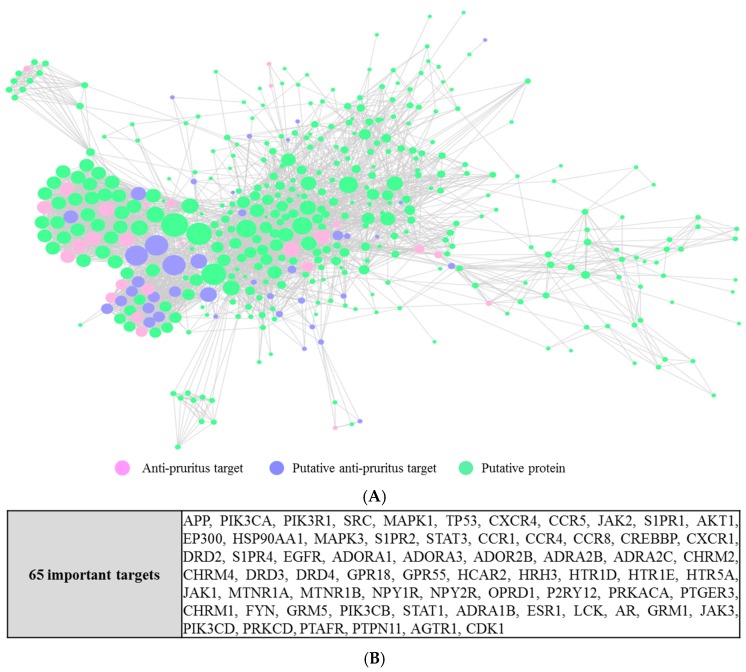
Protein–protein interaction (PPI) network of anti-pruritus targets and proteins that directly or indirectly interact with the targets. (**A**) The PPI network of anti-pruritus protein targets (The size of the circle represents the degree of a node); (**B**) A list of 65 important proteins which interact with the anti-pruritus targets.

We constructed a protein–protein interaction network from the 81 anti-pruritus targets and the 494 putative targets [38]. Our analysis resulted in a connected network of 451 proteins and 3782 interactions. From them, 66 were anti-pruritus targets, 385 were putative targets, and in the intersection lay 26 putative anti-pruritus targets. The detailed interactions are depicted in Figure 4A. With a hub node defined as a node with a degree greater than 2-fold of the average degree of all the nodes in the network [39] (the average degree of all the nodes in the network is 17 in our studies), 65 major hub nodes were identified (Figure 4B).

### 2.5. Functions of the 95 Putative Targets of RSF and FC

GO enrichment analysis on the 95 putative targets of RSF and FC (combining the 30 putative anti-pruritus targets with 65 important targets) indicated that they are involved in signal transduction, regulation, and response processes, such as intracellular signaling cascade (73.40%), second-messenger-mediated signaling (43.61%), and cell surface receptor-linked signal transduction (73.40%) (Table 1). Many targets are involved in G-protein coupled receptor (GPCR) signaling pathways. RSF and FC are targeting different proteins in the same pathway.

**Figure 5:**
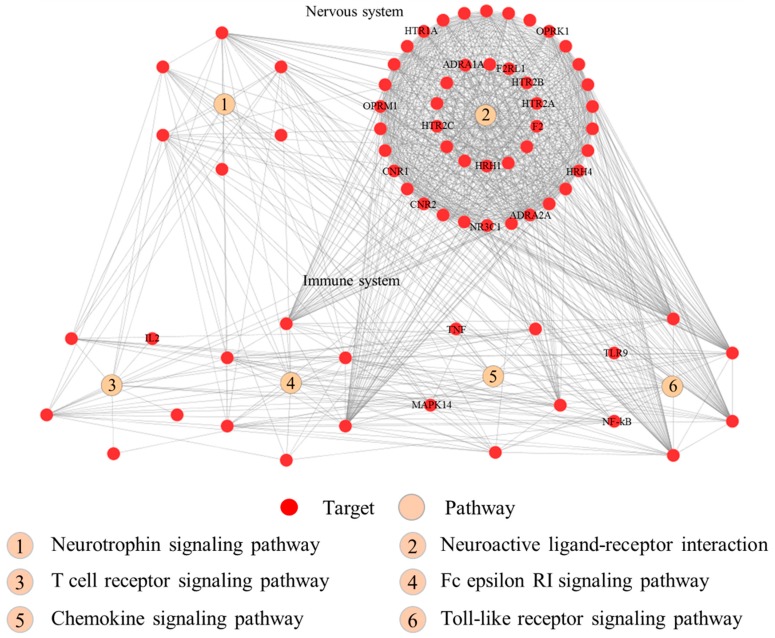
The interaction network of the proteins that are involved in the pruritus-related pathways.

The pathway enrichment analysis indicated that the 95 putative targets of RSF and FC were enriched in 46 Kyoto Encyclopedia of Genes and Genomes (KEGG) pathways (FDR corrected *p* value < 0.05). The top 20 pathways associated with the herbs are listed in the Table 2. Those pathways are divided into the following four groups: organismal system, environmental information processing, human disease, and cellular process. The four immune system pathways (toll-like receptor (TLR) signaling pathway, chemokine signaling pathway, T cell receptor signaling pathway, and Fc epsilon RI signaling pathway) and two nerve-related pathways (neurotrophin signaling pathway and neuroactive ligand-receptor interaction) are depicted in Figure 5.

Nine pathways are associated with both cancer (Table 2) and pruritus. It could explain how cancer patients often exhibit pruritus symptoms [40]; for example, a pancreatic cancer patient can have pruritus symptoms, which indicates the situation is getting worse [41]. Interleukin-2 (used to treat pruritus) can be used as a new therapy to cure renal carcinoma [42]. Prostate cancer patients often complain that the itching is the worst symptom [43].

**Figure 6:**
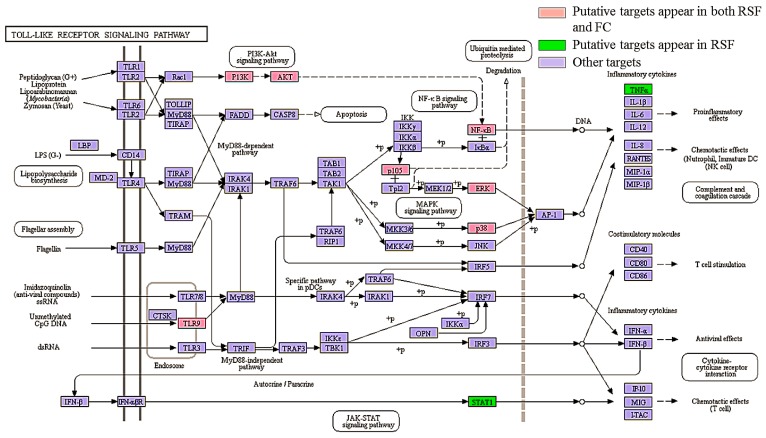
The participation of RSF and FC targets in the toll-like receptor signaling pathways.

The toll-like receptor signaling pathway involves pro-inflammatory cytokine production via activation of NF-κB and MAPK [44]. TLRs play an important role in the development and severity of pruritus by increasing the excitability of primary sensory neurons [45]. The RSF and FC putative targets were mapped to the TLR signaling pathway as shown in Figure 6. Seven putative targets are associated with both RSF and FC (highlighted in red). Two putative targets are associated with RSF (highlighted in green). These proteins regulate inflammation-related gene expression in the TLR signaling pathway.

## 3. Discussion

RSF–FC is a herb pair widely used for pruritus treatments. In this study, we elucidated the synergistic mechanism of this herb pair with data mining methods. The relationship between the herbs, ingredients, and targets of RSF and FC has been constructed. In RSF, alkaloids and flavonoids are the main active moieties. Matrine and oxymatrine (alkaloids) are known as anti-inflammatory, anti-allergic, anti-virus, anti-fibrotic, anti-tumor, and cardiovascular protective agents [46]. RSF flavonoids are known as anti-inflammatory and anti-arthritic [13] agents. FC coumarins are known as anti-pruritic, anti-allergic, and anti-inflammatory [17,18] agents.

By analyzing 30 putative anti-pruritus targets of RSF and FC, we found that the targets can be divided into the following two anti-pruritus target classes: (1) 17 nerve-related targets (such as serotonin receptors, opioid receptor, and transient receptor potential vanilloid receptor) and (2) 13 inflammation-related targets (such as cyclooxygenase, histamine receptors, cannabinoid receptors, and phosphodiesterase) [32]. Histamine H1 receptor (HRH1) plays a pivotal role in allergic inflammation and is considered a primary target for the pharmaceutical relief of pruritus [47]. Transient receptor potential vanilloid receptor 1 (TRPA1) is expressed in epidermal keratinocytes and in sensory neurons and plays an important role in pruritus pathogenesis [48]. Pretreatment with topical cannabinoid receptor agonists has been found to significantly reduce histamine-induced itch and vasodilatation in healthy volunteers [49]. Proteinase activated receptor 2 (F2RL1) is expressed in the epidermis of individuals with atopic dermatitis and plays an important role in pruritus pathogenesis [50].

By analyzing the PPI networks, we found 65 hub proteins that interact with the anti-pruritus targets. These 65 proteins can still be important for pruritus-related signal transduction. For example, signal transducer and activator of transcription 1 (STAT1) interacts with 5 proteins (interleukin-2 (IL2), mitogen-activated protein kinase 14 (MAPK14), nuclear factor kappa B p105 subunit (NF-κB), interleukin 6 (IL6), and mitogen-activated protein kinase 11(MAPK11)), of which four are related to anti-pruritus activity. NF-κB (which is associated with 5 putative anti-pruritus targets) is related to chronic inflammation and pathogenesis [51]. STAT1, IL2, and NF-κB are in the toll-like receptor signaling pathway and are related to pruritus pathogenesis [52].

Based on the functional analyses, we believe that compounds of RSF and FC may regulate at least 20 signal transduction pathways. The RSF–FC herb pair can regulate immune pathways (such as the TLR signaling pathway, chemokine signaling pathway, T cell receptor signaling pathway, and Fc epsilon RI signaling pathway) and nerve-related pathways (neurotrophin signaling pathway and neuroactive ligand-receptor interaction). TLRs are type I transmembrane receptors expressed in innate immune cells that detect pathogen infection or tissue damage. The activation of innate immune cells leads to the expression of inflammatory mediators such as cytokines, chemokines, leukotriene molecules, and histamine [52,53]. TLRs play an important role in the development and severity of pruritus by increasing the excitability of primary sensory neurons [45]. Chemokines are small chemoattractant peptides in cell trafficking, that are vital for a protective host response in an inflammatory immune response [54]. Chemokines are related to the development of atopic dermatitis itch [55]. The chemokine and TLR signaling pathways are associated with chronic mucocutaneous candidiasis (CMC), which is associated with pruritus and *Candida albicans* [56]. The neurotrophin signaling pathway is associated with acne inversa/hidradenitis suppurativa, which is also associated with pruritus [57]. Histamine receptors, opioid receptors, transient receptor potential vanilloid receptor, and cannabinoid receptors are pruritus pathogenesis modulators [32,33,34,35,36,37]. Mast cells can be activated and release biogenic amine granules (such as histamines) and proteoglycans (such as heparin) in the Fc epsilon RI signaling pathway [58]. The IgE receptor in myeloid dendritic cells also plays a major role in atopic dermatitis [59]. Based upon the above analyses, compounds of RSF and FC may regulate up to 20 signal transduction pathways. The RSF–FC herb pair can regulate immune pathways and nerve-related pathways.

## 4. Materials and Methods

### 4.1. Data Preparation

The chemical compound data derived from RSF and FC were collected from the Traditional Chinese Medicine Systems Pharmacology Database (TCMSP) [60] and other publications [26,27,61,62,63,64]. RSF includes 113 compounds, while FC includes 114 compounds. These compounds were clustered by a scaffold-based classification approach [65].

Anti-pruritus targets were derived from the Therapeutic Targets Database (TTD) [31] and literature [32,33,34,35,36,37].

Protein–protein interaction (PPI) data were derived from the Search Tool for the Retrieval of Interacting Genes (STRING) [38]. A protein–protein interaction was ranked with the three following confidence levels: low, scores < 0.4; medium, 0.4 to 0.7; and high, > 0.7. We only selected the high confidence scores (scores > 0.7) PPIs to construct the PPI network. There are 3782 PPIs with high confidence values (Supplementary Table S3).

### 4.2. Drug Targets Prediction for RSF and FC

Putative targets of RSF and FC were predicted based on molecular scaffolds which derived from the active components of RSF and FC. The known putative targets of RSF and FC were identified from the Annotated Scaffold Database (ASDB) [25]. The putative targets of other molecular scaffolds which don’t have known putative targets were predicted by the Pharmaceutical Target Seeker (PTS, http://www.rcdd.org.cn/PTS/index) with a 3D structure similarity threshold of 90% and a ligand activity threshold (IC_50_/Ki/Kd) <10 μM.

### 4.3. Network Construction and Network Analysis

A herb-scaffold-target network consists of the herbs, molecular scaffolds, and putative targets. The network was constructed based on the data from Section 4.1 and Section 4.2. Based on the PPI data in Section 4.1, we constructed a PPI network for the putative target proteins of herbs and the anti-pruritus target proteins to obtain the hub effective proteins. The networks were visualized with Cytoscape [66].

A network node degree is the number of edges connected to the node. A node with a higher degree (hub node) is more important in the network. A hub node degree should be two times greater than the average node degree in the network [39].

### 4.4. Gene Ontology and Pathway Enrichment Analysis

The Database for Annotation, Visualization and Integrated Discovery (DAVID) [67] was used to perform Gene Ontology (GO) enrichment analysis for putative targets targeted by RSF and FC. DAVID was also employed to conduct pathway enrichment analysis for the same gene products to identify the potential synergistic effects of RSF and FC against pruritus. *Homo sapiens* was selected as the species, and the default background was the whole genome of *Homo sapiens*. Enriched GO terms and pathways were defined as those with adjusted *p*-values < 0.05.

## 5. Conclusions

Our studies demonstrate that the mechanisms of action of the RSF–FC herbal pair can be elucidated through data mining approaches. Using the chemical structures of the compounds derived from RSF and FC, we were able to predict RSF and FC targets. By mapping the putative targets onto a PPI network, we found more proteins that interact with the putative anti-pruritus targets for RSF and FC. By examining the biological function annotations of these proteins, we learned that; (1) The active compounds of RSF are mainly alkaloids and flavonoids. The alkaloids can be targeting inflammation-related proteins (MAPK14, PTGS2, PTGS1, and F2) and pruritus-related proteins (HRH1, TRPA1, HTR3A, and HTR6). The representative putative targets of the flavonoids are inflammation-related proteins (TNF, NF-κB, F2, PTGS2, and PTGS1) and pruritus-related proteins (NR3C1 and IL2); (2) The active compounds of FC are mainly coumarins, which target CNS-related proteins (AChE and OPRK1) and inflammation-related proteins (PDE4D, TLR9, and NF-κB); (3) Both RSF and FC have anti-inflammatory effects, though they exhibit anti-pruritus effects in different ways.

## Figures and Tables

**Table 1:** The top 15 biological processes enriched by the 95 putative targets.

Term	Count of Proteins	%	Proteins Associate with RSF	Proteins Associate with FC	*p*-Value
Intracellular signaling cascade	69	73.40	57	56	1.05 × 10^−50^
Second-messenger-mediated signaling	41	43.61	31	33	1.09 × 10^−46^
Cell surface receptor linked signal transduction	69	73.40	55	56	1.97 × 10^−39^
G-protein coupled receptor protein signaling pathway	55	58.51	41	45	5.81 × 10^−35^
Cyclic-nucleotide-mediated signaling	27	28.72	18	24	8.46 × 10^−32^
G-protein signaling, coupled to cyclic nucleotide second messenger	26	27.65	17	23	1.48 × 10^−31^
Positive regulation of catalytic activity	36	38.29	34	28	3.49 × 10^−26^
Positive regulation of molecular function	37	39.36	34	29	1.32 × 10^−25^
Protein kinase cascade	31	32.97	28	26	1.21 × 10^−24^
behavior	32	34.04	28	28	8.15 × 10^−23^
CAMP-mediated signaling	18	19.14	13	17	4.94 × 10^−20^
G-protein signaling, coupled to cAMP nucleotide second messenger	17	18.08	12	16	2.82 × 10^−19^
Cellular calcium ion homeostasis	21	22.34	19	17	5.05 × 10^−19^
Calcium ion homeostasis	21	22.34	19	17	8.69 × 10^−19^
Cellular metal ion homeostasis	21	22.34	19	17	2.00 × 10^−18^

**Table 2:** The top 20 KEGG pathways of 95 putative targets with Benjamini Hochberg corrected *p*-values < 0.05 generated by DAVID.

KEGG Pathway	Count of Proteins	Proteins Associate with RSF	Proteins Associate with FC	*p*-Value	Class
Toll-like receptor signaling pathway	12	12	10	8.77 × 10^−7^	Organismal Systems; Immune system
Chemokine signaling pathway	20	20	16	1.76 × 10^−10^	Organismal Systems; Immune system
T cell receptor signaling pathway	13	13	11	2.13 × 10^−7^	Organismal Systems; Immune system
Fc epsilon RI signaling pathway	11	11	9	6.19 × 10^−7^	Organismal Systems; Immune system
Neurotrophin signaling pathway	12	12	11	6.77 × 10^−6^	Organismal Systems; Nervous system
Progesterone-mediated oocyte maturation	11	11	11	1.56 × 10^−6^	Organismal Systems; Endocrine system
Neuroactive ligand-receptor interaction	44	31	37	8.44 × 10^−34^	Environmental Information Processing; Signaling molecules and interaction
Calcium signaling pathway	17	15	12	2.98 × 10^−8^	Environmental Information Processing; Signal transduction
Jak-STAT signaling pathway	14	13	10	1.73 × 10^−6^	Environmental Information Processing; Signal transduction
VEGF signaling pathway	10	10	10	4.13 × 10^−6^	Environmental Information Processing; Signal transduction
Pancreatic cancer	13	13	11	1.94 × 10^−9^	Human Diseases; Cancers
Prostate cancer	14	13	14	2.11 × 10^−9^	Human Diseases; Cancers
Endometrial cancer	9	9	9	2.16 × 10^−6^	Human Diseases; Cancers
Renal carcinoma	10	9	10	2.29 × 10^−6^	Human Diseases; Cancers
Non-small cell lung cancer	9	9	9	2.91 × 10^−6^	Human Diseases; Cancers
Chronic myeloid leukemia	10	10	10	4.13 × 10^−6^	Human Diseases; Cancers
Acute myeloid leukemia	9	9	9	5.06 × 10^−6^	Human Diseases; Cancers
Pathways in cancer	19	18	16	7.50 × 10^−6^	Human Diseases; Cancers
Glioma	9	9	9	9.54 × 10^−6^	Human Diseases; Cancers

## Supplementary Materials

Supplementary Materials are available online.

## References

[1] Yosipovitch G., Greaves M.W., Schmelz M. (2003). Itch. Lancet.

[2] Hanifin J.M., Ling M.R., Langley R., Breneman D., Rafal E. (2001). Tacrolimus ointment for the treatment of atopic dermatitis in adult patients: Part I, efficacy. J. Am. Acad. Dermatol..

[3] Jekler J., Larkö O. (1990). Combined UVA-UVB versus UVB phototherapy for atopic dermatitis a paired-comparison study. J. Am. Acad. Dermatol..

[4] Wahlgren C., Scheynius A., Hägermark Ö. (1990). Antipruritic effect of oral cyclosporin A in atopic dermatitis. Acta Derm. Venereol..

[5] Draelos Z.D. (2008). Use of topical corticosteroids and topical calcineurin inhibitors for the treatment of atopic dermatitis in thin and sensitive skin areas. Curr. Med. Res. Opin..

[6] Zhao J., Jiang P., Zhang W. (2010). Molecular networks for the study of TCM pharmacology. Brief. Bioinform..

[7] Wang S., Hu Y., Tan W., Wu X., Chen R., Cao J., Chen M., Wang Y. (2012). Compatibility art of traditional Chinese medicine: From the perspective of herb pairs. J. Ethnopharmacol..

[8] Ung C.Y., Li H., Cao Z.W., Li Y.X., Chen Y.Z. (2007). Are herb-pairs of traditional Chinese medicine distinguishable from others? Pattern analysis and artificial intelligence classification study of traditionally defined herbal properties. J. Ethnopharmacol..

[9] Liu J., Zhu M., Shi R., Yang M. (2003). *Radix Sophorae flavescentis* for Chronic Hepatitis B A Systematic Review of Randomized Trials. Am. J. Chin. Med..

[10] Yang X., Cai W., Yang Q., Lu Z., Li J., Yu J. (2015). Compound *Radix Sophorae Flavescentis* exerts antitumor effects by inhibiting the proliferation and inducing the apoptosis of esophageal carcinoma TE-8 cells. Oncol. Lett..

[11] Drew A.K., Bensoussan A., Whyte I.M., Dawson A.H., Zhu X., Myers S.P. (2002). Chinese herbal medicine toxicology database monograph on *Radix Sophorae Flavescentis*, ku shen. Clin. Toxicol..

[12] Hwang G.B., Lee J.E., Nho C.W., Lee B.U., Lee S.J., Jung J.H., Bae G.N. (2012). Short-term effect of humid airflow on antimicrobial air filters using Sophora flavescens nanoparticles. Sci. Total Environ..

[13] Jin J.H., Kim J.S., Kang S.S., Son K.H., Chang H.W., Kim H.P. (2010). Anti-inflammatory and anti-arthritic activity of total flavonoids of the roots of Sophora flavescens. J. Ethnopharmacol..

[14] Xiao Z.M., Wang A.M., Wang X.Y., Shen S.R. (2013). Effects of ethanol extract of *Radix Sophorae Flavescentis* on activity of colon cancer HT29 cells. Afr. J. Tradit. Complement. Altern. Med..

[15] Xu W., Lin H., Zhang Y., Chen X., Hua B., Hou W., Qi X., Pei Y., Zhu X., Zhao Z. (2011). Compound Kushen Injection suppresses human breast cancer stem-like cells by down-regulating the canonical Wnt/β-catenin pathway. J. Exp. Clin. Cancer Res..

[16] Wagner H., Bauer R., Melchart D., Xiao P.G., Staudinger A. (2011). Chromatographic Fingerprint Analysis of Herbal Medicines.

[17] Mastuda H., Tomohiro N., Ido Y., Kubo M. (2002). Anti-allergic effects of cnidii monnieri fructus (dried fruits of Cnidium monnieri) and its major component, osthol. Biol. Pharm. Bull..

[18] Matsuda H., Ido Y., Hirata A., Ino Y., Naruto S., Amamiya T., Kuboa M. (2002). Antipruritic effect of Cnidii Monnieri Fructus (fruits of Cnidium monnieri CUSSON). Biol. Pharm. Bull..

[19] Mouduo L., Cuixia Q., Liping Q., Junyong Z., Changquan L. (2012). Application of traditional Chinese medicine injection in treatment of primary liver cancer: A review. J. Tradit. Chin. Med..

[20] Hopkins A.L. (2007). Network pharmacology. Nat. Biotechnol..

[21] Wen Z., Wang Z., Wang S., Ravula R., Yang L., Xu J., Wang C., Zuo Z., Chow M.S., Shi L. (2011). Discovery of molecular mechanisms of traditional Chinese medicinal formula Si-Wu-Tang using gene expression microarray and connectivity map. PLoS ONE.

[22] Zhang Y., Guo X., Wang D., Li R., Li X., Xu Y., Liu Z., Song Z., Lin Y., Li Z. (2014). A systems biology-based investigation into the therapeutic effects of Gansui Banxia Tang on reversing the imbalanced network of hepatocellular carcinoma. Sci. Rep..

[23] Zhang Y., Bai M., Zhang B., Liu C., Guo Q., Sun Y., Wang D., Wang C., Jiang Y., Lin N. (2015). Uncovering pharmacological mechanisms of Wu-tou decoction acting on rheumatoid arthritis through systems approaches: Drug-target prediction, network analysis and experimental validation. Sci. Rep..

[24] Li J., Zhao P., Li Y., Tian Y., Wang Y. (2015). Systems pharmacology-based dissection of mechanisms of Chinese medicinal formula Bufei Yishen as an effective treatment for chronic obstructive pulmonary disease. Sci. Rep..

[25] Liu Z., Ding P., Yan X., Zheng M., Zhou H., Xu Y., Du Y., Gu Q., Xu J. (2016). ASDB: A resource for probing protein functions with small molecules. Bioinformatics.

[26] Li W., Liang H., Yin T., Wang B., Zhao Y.Y. (2008). Main flavonoids from Sophora flavescenes. Yao Xue Xue Bao.

[27] Li S., Wang S. (1999). Determination of alkaloids in *Radix Sophorae Flavescentis* by high performance capillary electrophoresis. Zhongguo Zhong Yao Za Zhi.

[28] Davalos D., Baeten K.M., Whitney M.A., Mullins E.S., Friedman B., Olson E.S., Ryu J.K., Smirnoff D.S., Petersen M.A., Bedard C. (2014). Early detection of thrombin activity in neuroinflammatory disease. Ann. Neurol..

[29] Nair S., Doh S.T., Chan J.Y., Kong A.N., Cai L. (2008). Regulatory potential for concerted modulation of Nrf2- and Nfkb1-mediated gene expression in inflammation and carcinogenesis. Br. J. Cancer.

[30] Willoughby D.A., Moore A.R., Colville-Nash P.R. (2000). COX-1, COX-2, and COX-3 and the future treatment of chronic inflammatory disease. Lancet.

[31] Chen X., Ji Z.L., Chen Y.Z. (2002). TTD Therapeutic Target Database. Nucleic Acids Res..

[32] Potenzieri C., Undem B.J. (2012). Basic mechanisms of itch. Clin. Exp. Allergy.

[33] Ong P.Y. (2009). Emerging Drugs for atopic dermatitis. Expert Opin. Emerg. Drugs.

[34] Benecke H., Lotts T., Ständer S. (2013). Investigational drugs for pruritus. Expert Opin. Investig. Drugs.

[35] Ständer S., Luger T.A. (2010). Itch in Atopic Dermatitis—Pathophysiology and Treatment. Acta Dermatovenerol. Croat..

[36] Davidson S., Giesler G.J. (2010). The multiple pathways for itch and their interactions with pain. Trends Neurosci..

[37] Liu T., Ji R.R. (2013). New insights into the mechanisms of itch: Are pain and itch controlled by distinct mechanisms?. Pflugers Arch..

[38] Szklarczyk D., Franceschini A., Kuhn M., Simonovic M., Roth A., Minguez P., Doerks T., Stark M., Muller J., Bork P. (2011). The STRING database in 2011: Functional interaction networks of proteins, globally integrated and scored. Nucleic Acids Res..

[39] Li S., Zhang Z.Q., Wu L.J., Zhang X.G., Wang Y.Y., Li Y.D. (2007). Understanding ZHENG in traditional Chinese medicine in the context of neuro-endocrine-immune network. IET Syst. Biol..

[40] Lidstone V., Thorns A. (2001). Pruritus in cancer patients. Cancer Treat. Rev..

[41] Owens D.J., Savides T.J. (2010). Endoscopic ultrasound staging and novel therapeutics for pancreatic cancer. Surg. Oncol. Clin. N. Am..

[42] Lee S., Dasanu C., Dutcher J. (2006). Eosinophilia and Pruritus With Interleukin-2 Treatment in Patients With Metastatic Renal Cell Carcinoma. J. Immunother..

[43] Bosonnet L. (2003). Pruritus: Scratching the surface. Eur. J. Cancer Care.

[44] Kawai T., Akira S. (2007). TLR signaling. Semin. Immunol..

[45] Tong L., Yong-Jing G., Rong J.R. (2012). Emerging role of toll-like receptors in the control of pain and itch. Neurosci. Bull..

[46] Liu Y., Xu Y., Ji W., Li X., Sun B., Gao Q., Su C. (2014). Anti-tumor activities of matrine and oxymatrine: Literature review. Tumour Biol..

[47] Simons F.E. (2004). Advances in H1-antihistamine. N. Engl. J. Med..

[48] Wilson S.R., Gerhold K.A., Bifolck-Fisher A., Liu Q., Patel K.N., Dong X., Bautista D.M. (2011). TRPA1 is required for histamine-independent, Mas-related G protein-coupled receptor-mediated itch. Nat. Neurosci..

[49] Dvorak M., Watkinson A., McGlone F., Rukwied R. (2003). Histamine induced responses are attenuated by a cannabinoid receptor agonist in human skin. Inflamm. Res..

[50] Steinhoff M., Neisius U., Ikoma A., Fartasch M., Heyer G., Skov P.S., Luger T.A., Schmelz M. (2008). Proteinase-activated receptor-2 mediates itch: A novel pathway for pruritus in human skin. Exp. Dermatol..

[51] Tenda Y., Yamashita M., Kimura M.Y., Hasegawa A., Shimizu C., Kitajima M., Onodera A., Suzuki A., Seki N., Nakayama T. (2006). Hyperresponsive TH2 cells with enhanced nuclear factor-kappa B activation induce atopic dermatitis-like skin lesions in Nishiki-nezumi Cinnamon/Nagoya mice. J. Allergy Clin. Immunol..

[52] Vabulas R.M., Ahmad-Nejad P., da Costa C., Miethke T., Kirschning C.J., Hacker H., Wagner H. (2001). Endocytosed HSP60s use toll-like receptor 2 (TLR2) and TLR4 to activate the toll/interleukin-1 receptor signaling pathway in innate immune cells. J. Biol. Chem..

[53] Kawasaki T., Kawai T. (2014). Toll-like receptor signaling pathways. Front. Immunol..

[54] Wong M.M., Fish E.N. (2003). Chemokines attractive mediators of the immune response. Semin. Immunol..

[55] Uchida T., Suto H., Ra C., Ogawa H., Kobata T., Okumura K. (2002). Preferential expression of Th2-type chemokine and its receptor in atopic dermatitis. Int. Immunol..

[56] Veraldi S. (2013). Rapid relief of intertrigo-associated pruritus due to Candida albicans with isoconazole nitrate and diflucortolone valerate combination therapy. Mycoses.

[57] Mônica Mourthé de Alvim A., José Roberto Monteiro C., Daniel Martins Barbosa M.G., Flávia Fontes F., Rodrigo Guimarães O., Renata Magali R., Silluzio F., Geraldo Magela Gomes da C. (2012). Hidradenitis suppurativa literature review and case report. J. Coloproctol..

[58] Tkaczyk C., Gilfillan A.M. (2001). Fc(epsilon)Ri-dependent signaling pathways in human mast cells. Clin. Immunol..

[59] Novak N., Allam J.P., Hagemann T., Jenneck C., Laffer S., Valenta R., Kochan J., Bieber T. (2004). Characterization of FcepsilonRI-bearing CD123 blood dendritic cell antigen-2 plasmacytoid dendritic cells in atopic dermatitis. J. Allergy Clin. Immunol..

[60] Ru J., Li P., Wang J., Zhou W., Li B., Huang C., Li P., Guo Z., Tao W., Yang Y. (2014). TCMSP a database of systems pharmacology for drug discovery from herbal medicines. J. Cheminform..

[61] Weng D.X., Li T.A. (2007). Simultaneous determination of 5 active components in *Fructus Cnidii* by HPLC. Zhongguo Zhong Yao Za Zhi.

[62] Jiang Y.Q. (2006). Analysis of coumarins in *Fructus Cnidii* by HPLC-ESI-MS. Zhong Yao Cai.

[63] Wu M.H., Zhao L.H., Song Y., Zhang W., Xiang B.R., Mei L.H. (2005). Determination of five coumarins in Cnidii Fructus by micellar electrokinetic capillary chromatography. Planta Med..

[64] Sun W.J., Sha Z.F., Gao H. (1990). Determination of osthol and imperatorin in *Cnidium monnieri* (L.) cuss by fluorometry TLC scanning. Yao Xue Xue Bao.

[65] Xu J. (2002). A New Approach to Finding Natural Chemical Structure Classes. J. Med. Chem..

[66] Shannon P., Markiel A., Ozier O., Baliga N.S., Wang J.T., Ramage D., Amin N., Schwikowski B., Ideker T. (2003). Cytoscape: A Software Environment for Integrated Models of Biomolecular Interaction Networks. Genome Res..

[67] Huang da W., Sherman B.T., Lempicki R.A. (2009). Systematic and integrative analysis of large gene lists using DAVID bioinformatics resources. Nat. Protocol..

