# Supplementary material for: Synergic Anti-Pruritus Mechanisms of Action for the Radix Sophorae Flavescentis and Fructus Cnidii Herbal Pair
Source: Molecules. 2017 Sep 4;22(9):1465. doi: 10.3390/molecules22091465 (PMC6151778; doi:10.3390/molecules22091465)
Supplement: Supplementary file 1 [file molecules-22-01465-s001.zip › Supplementary Files/Supplementary Table S1.docx]

**Supplementary Table S1: The candidate scaffolds and** **their corresponding compounds**

| Herb | Scaffold | Ingredients | Count of targets |
| --- | --- | --- | --- |
| Radix Sophorae Flavescentis |  | 2-n-heptadecyl-5,7-dihydroxy-6,8-dimethyl chromone | 48 |
| Radix Sophorae Flavescentis |  | (+)-lehmannine | 58 |
| Radix Sophorae Flavescentis |  | sophocarpine; 9alpha-hydroxy-13,14-didehydromatridine-15-one; n-oxysophocarpine; oxysophocarpine; (-)-9alpha-hydroxysophoramine; isosophocarpine | 62 |
| Radix Sophorae Flavescentis |  | kushenol e; naringenin; cis-dihydroquercetin; liquiritigenin; hesperetin; kushenol m; (2r)-flavanone; isokurarinone; isoxanthohumol; (-)-kurarinone; kurarinol; kushenol a; kushenol b; kushenol f; kushenol j; kushenol n; kushenol t; Leachianone a; Leachianone g; sophoraflavanone g; nymphaeol a; kosamol,q; kosamol,r; kushenol J_qt; sokurarinone | 62 |
| Radix Sophorae Flavescentis |  | 2-hydroxychalcone; kuraridinol; kuraridin; kushenol d; xanthohumol; 1-[2,4-dihydroxy-3-(3-methylbut-2-enyl)phenyl]-3-phenylprop-2-en-1-one; (E)-1-(2,6-dihydroxyphenyl)-3-(4-hydroxyphenyl)prop-2-en-1-one; kurarinone; sophoraflavanone,g | 76 |
| Radix Sophorae Flavescentis |  | cid 5316459; 7,11-dehydromatrine; 9alpha-hydroxy-7,11-dehydromatrine | 78 |
| Radix Sophorae Flavescentis |  | kuraridine | 84 |
| Radix Sophorae Flavescentis |  | sophoridine; matrine; (+)-14alpha-hydroxymatrine; (+)-9alpha-hydroxymatrine; (+)-allomatrine; sophoranol; (-)-14beta-hydroxymatrine; 5,9-dihydroxymatrine; (-)-allomatrine; oxymatrine; sophoranol n-oxide; sophranol; AIDS211310 | 84 |
| Radix Sophorae Flavescentis |  | formononetin; wighteone | 85 |
| Radix Sophorae Flavescentis |  | sophoramine | 97 |
| Radix Sophorae Flavescentis |  | 1,4-diazaindan-type,alkaloid,flavascensine | 109 |
| Radix Sophorae Flavescentis |  | 7-demethylsuberosin; 7-prenyloxycoumarin | 186 |
| Radix Sophorae Flavescentis |  | luteolin; apigenin; quercetin; ac1nsxey; kushenol g; 8-Isopentenyl-kaempferol; 3,4',5-Trihydroxy-7-methoxy-8-isopente-nylflavone; Norartocarpetin | 336 |
| Fructus Cnidii |  | cnideol B | 46 |
| Fructus Cnidii |  | muurolene; alpha cadinene | 63 |
| Fructus Cnidii |  | cis-thujopsene | 68 |
| Fructus Cnidii |  | osthol; 7-methoxy-4-methylcoumarin; paniculal; auraptenol; isomexoticin; ostruthin | 186 |
| Fructus Cnidii |  | isobutyrylshikonin; isobutylalkannin | 77 |
| Fructus Cnidii |  | (+)-gamma-muurolene | 93 |
| Fructus Cnidii |  | cnidimol f | 100 |
| Fructus Cnidii |  | cnidilide | 103 |
| Fructus Cnidii |  | cnidimol b | 194 |
| Fructus Cnidii |  | diosmetin; | 336 |
| Fructus Cnidii |  | cnidimol c; cnidimol d; cnidimol A; cnidimol E; | 48 |
