# Supplementary material for: Synergic Anti-Pruritus Mechanisms of Action for the Radix Sophorae Flavescentis and Fructus Cnidii Herbal Pair
Source: Molecules. 2017 Sep 4;22(9):1465. doi: 10.3390/molecules22091465 (PMC6151778; doi:10.3390/molecules22091465)
Supplement: Supplementary file 1 [file molecules-22-01465-s001.zip › Supplementary Files/Supplementary Table S2.docx]

**Supplementary Table S2: Known anti-pruritus targets**

| Name | Uniport | Gene name | Reference |
| --- | --- | --- | --- |
| 1-phosphatidylinositol 4,5-bisphosphate phosphodiesterase beta-3 | Q01970 | PLCB3 | Ref5/Ref6 |
| 5-hydoxytryptamine 2A receptor | P28223 | HTR2A | Ref[1](#_ENREF_1)/Ref6 |
| 5-hydoxytryptamine 2B receptor | P41595 | HTR2B | Ref[1](#_ENREF_1)/Ref6 |
| 5-hydoxytryptamine 2C receptor | P28335 | HTR2C | Ref[1](#_ENREF_1)/Ref6 |
| 5-hydroxytryptamine 6 receptor | P50406 | HTR6 | TTD |
| 5-hydroxytryptamine receptor 3A | P46098 | HTR3A | TTD |
| Acetylcholinesterase | P22303 | ACHE | Ref[1](#_ENREF_1)/Ref7 |
| Alpha-1A adrenergic receptor | P35348 | ADRA1A | Ref4 |
| Alpha-2A adrenergic receptor | P08913 | ADRA2A | Ref4 |
| Beta-defensin 1 | P60022 | DEFB1 | Ref2 |
| Bile acid receptor | Q96RI1 | NR1H4 | Ref6 |
| Calcium-activated potassium channel | Q12791 | KCNMA1 | Ref[1](#_ENREF_1)/Ref3 |
| cAMP-specific 3',5'-cyclic phosphodiesterase 4B | Q07343 | PDE4B | Ref2 |
| cAMP-specific 3',5'-cyclic phosphodiesterase 4D | Q08499 | PDE4D | Ref2 |
| Cannabinoid receptor1 | P21554 | CNR1 | Ref2/Ref4/Ref7 |
| Cannabinoid receptor2 | P34972 | CNR2 | Ref2/Ref4/Ref7 |
| Chymase | P23946 | CMA1 | Ref2 |
| Cyclooxygenase-1 | P23219 | PTGS1 | Ref2 |
| Cysteinyl leukotriene receptor 2 | Q9NS75 | CYSLTR2 | Ref[1](#_ENREF_1)/Ref4 |
| Cysteinyl leukotriene receptor1 | Q9Y271 | CYSLTR1 | Ref[1](#_ENREF_1)/Ref4 |
| Dihydrofolate reductase | P00374 | DHFR | Ref5 |
| Endothelin-1 | P05305 | EDN1 | Ref6 |
| Fatty-acid amide hydrolase 1 | O00519 | FAAH | Ref7 |
| Fatty-acid amide hydrolase 2 | Q6GMR7 | FAAH2 | Ref7 |
| Fibroblast growth factor receptor 2 | P21802 | FGFR2 | Ref[1](#_ENREF_1)/Ref3/Ref4/Ref6/Ref7 |
| Filaggrin | P20930 | FLG | Ref2 |
| Gastrin-releasing peptide receptor | P30550 | GRPR | Ref3/Ref5/Ref7 |
| Glucocorticoid receptor | P04150 | NR3C1 | Ref2 |
| High affinity nerve growth factor receptor | P04629 | NTRK1 | Ref3/Ref4/Ref6/Ref7 |
| Histamine 4 receptor | Q9H3N8 | HRH4 | Ref[1](#_ENREF_1)/Ref3/Ref4/Ref5/Ref6/Ref7 |
| Histamine H1 receptor | P35367 | HRH1 | TTD/Ref1/Ref3/Ref4/Ref5/Ref6/Ref7 |
| histidine decarboxylase | P19113 | HDC | Ref6 |
| Interferon-gamma | P01579 | IFNG | Ref2/Ref6 |
| Interleukin 13 | P35225 | IL13 | Ref2 |
| Interleukin 2 | P60568 | IL2 | Ref4/Ref6 |
| Interleukin 4 | P05112 | IL4 | Ref2 |
| Interleukin 6 | P05231 | IL6 | Ref[1](#_ENREF_1)/Ref4 |
| Interleukin 8 | P10145 | IL8 | Ref4 |
| Interleukin-17 receptor C | Q8NAC3 | IL17RC | Ref2 |
| Interleukin-31 receptor subunit alpha | Q8NI17 | IL31RA | Ref[1](#_ENREF_1)/Ref3/ Ref2/Ref4/ Ref6/Ref7 |
| Interleukin-4 receptor subunit alpha | P24394 | IL4R | Ref2 |
| involucrin | P07476 | IVL | Ref2 |
| Kappa-type opioid receptor | P41145 | OPRK1 | Ref3/Ref7 |
| Leukotriene B4 receptor 1 | Q15722 | LTB4R | Ref[1](#_ENREF_1)/Ref6 |
| Leukotriene B4 receptor 2 | Q9NPC1 | LTB4R2 | Ref[1](#_ENREF_1)/ Ref6 |
| Lysophosphatidic acid receptor 1 | Q92633 | LPAR1 | Ref[1](#_ENREF_1)/Ref6/Ref7 |
| Lysophosphatidic acid receptor 2 | Q9HBW0 | LPAR2 | Ref[1](#_ENREF_1)/Ref6/Ref7 |
| Lysophosphatidic acid receptor 3 | Q9UBY5 | LPAR3 | Ref[1](#_ENREF_1)/Ref6/Ref7 |
| Lysophosphatidic acid receptor 6 | P43657 | LPAR6 | Ref[1](#_ENREF_1)/Ref6/Ref7 |
| MAS related GPR family member D | Q8TDS7 | MRGPRD | Ref6 |
| MAS-related GPR, member X1 | Q96LB2 | MRGPRX1 | Ref6 |
| Mitogen-activated protein kinase 11 | Q15759 | MAPK11 | Ref6 |
| Mitogen-activated protein kinase 14 | Q16539 | MAPK14 | Ref6 |
| Mu-type opioid receptor | P35372 | OPRM1 | Ref3/Ref6/Ref7 |
| Neurotrophin 4 | P34130 | NTF4 | Ref[1](#_ENREF_1)/Ref4 |
| Nuclear factor NF-kappa-B p105 subunit | P19838 | NFKB1 | Ref2/Ref4 |
| Nucleotide-binding oligomerization domain-containing protein 1 | Q9Y239 | NOD1 | Ref2 |
| Peroxisome proliferator-activated receptor gamma | P37231 | PPARG | Ref2 |
| Phospholipase A2 | P04054 | PLA2G1B | Ref5/Ref7 |
| Prostaglandin D2 receptor 2 | Q9Y5Y4 | PTGDR2 | Ref2 |
| Prostaglandin G/H synthase | P35354 | PTGS2 | Ref2 |
| Protease-activated receptors 4 | Q96RI0 | F2RL3 | Ref[1](#_ENREF_1)/Ref5/Ref6/Ref7 |
| Proteinase activated receptor 2 | P55085 | F2RL1 | TTD/Ref[1](#_ENREF_1)/Ref2/Ref5/Ref6/Ref7 |
| Semaphorin 3A | Q14563 | SEMA3A | Ref2/Ref3 |
| Serine/threonine-protein kinase PAK 2 | Q13177 | PAK2 | Ref6 |
| Serotonin 1a (5-HT1a) receptor | P08908 | HTR1A | Ref[1](#_ENREF_1) |
| Sphingosine kinase 1 | Q9NYA1 | SPHK1 | Ref2 |
| Substance-K receptor | P21452 | TACR2 | Ref[1](#_ENREF_1) |
| Substance-P receptorMDC | P25103 | TACR1 | Ref[1](#_ENREF_1)/Ref3/Ref4/Ref5/Ref7 |
| TGF-beta-activated kinase 1 and MAP3K7-binding protein 1 | Q15750 | TAB1 | Ref2 |
| Thrombin | P00734 | F2 | Ref2 |
| TNF-alpha | P01375 | TNF | Ref6 |
| Toll like receptor 2 | O60603 | TLR2 | Ref5 |
| Toll like receptor 3 | O15455 | TLR3 | Ref6 |
| Toll like receptor 4 | O00206 | TLR4 | Ref6 |
| Toll like receptor 7 | Q9NYK1 | TLR7 | Ref[1](#_ENREF_1)/Ref6/Ref7 |
| Toll-like receptor 9 | Q9NR96 | TLR9 | Ref5 |
| Transient receptor potential cation channel subfamily A member 1 | O75762 | TRPA1 | Ref3/Ref5/Ref6 |
| Transient receptor potential cation channel subfamily M member 8 | Q7Z2W7 | TRPM8 | Ref3 |
| transient receptor potential cation channel subfamily V member 1 | Q8NER1 | TRPV1 | TTD/Ref[1](#_ENREF_1)/Ref3/Ref4/Ref5/Ref6/Ref7 |
| Transient receptor potential cation channel subfamily V member 3 | Q8NET8 | TRPV3 | Ref3/ Ref7 |

# Reference

1. Potenzieri, C.; Undem, B. J., Basic mechanisms of itch. *Clinical and experimental allergy : journal of the British Society for Allergy and Clinical Immunology* **2012,** *42* (1), 8-19.

2. Ong, P. Y., Emerging Drugs for atopic dermatitis. *Expert Opin* **2009,** *14* (1).

3. Benecke, H.; Lotts, T.; Sta¨nder, S., Investigational drugs for pruritus. *Expert Opin* **2013,** *22* (9).

4. Ständer, S.; Luger, T. A., Itch in Atopic Dermatitis – Pathophysiology and Treatment. *18* **2010,** *Acta Dermatovenerol Croat* (4), 289-96.

5. Davidson, S.; Giesler, G. J., The multiple pathways for itch and their interactions with pain. *Trends in neurosciences* **2010,** *33* (12), 550-8.

6. Liu, T.; Ji, R. R., New insights into the mechanisms of itch: are pain and itch controlled by distinct mechanisms? *Pflugers Archiv : European journal of physiology* **2013,** *465* (12), 1671-85.

7. Tey, H. L.; Yosipovitch, G., Targeted treatment of pruritus: a look into the future. *The British journal of dermatology* **2011,** *165* (1), 5-17.
